# Virtually Wall-Less versus Standard Thin-Wall Venous Cannula in the Minimally Invasive Mitral Valve Surgery: Single-Center Experience

**DOI:** 10.3390/medicina59071221

**Published:** 2023-06-29

**Authors:** Fabrizio Ceresa, Liborio Francesco Mammana, Aurora Leonardi, Augusto Palermo, Francesco Patanè

**Affiliations:** Cardio-Vascular and Thoracic Department, Papardo Hospital, 98121 Messina, Italy; liborio.mammana@gmail.com (L.F.M.); auroraleonardi38@yahoo.it (A.L.); augustopalermo@aopapardo.it (A.P.); f_patane@hotmail.com (F.P.)

**Keywords:** minimally invasive mitral valve surgery, virtually wall-less cannula

## Abstract

*Background and Objectives*: Minimally invasive cardiac surgery (MICS) has been developing since 1996. Peripheral cannulation is required to perform MICS, and good venous drainage and a bloodless field are crucial for the success of this procedure. We assessed the benefits of using a virtually wall-less cannula in comparison with the standard thin-wall cannula in clinical practice. *Materials and Methods*: Between January 2021 and December 2022, we evaluated 65 elective patients, who underwent isolated minimally invasive mitral valve surgery. Both the virtually wall-less and the thin-wall cannulas were placed through a surgical cut-down. Patients’ characteristics at baseline were similar in the two groups, except for the body surface area (BSA), which was greater in the virtually wall-less group compared to the thin-wall one. In the standard group, the size of the cannula was chosen depending on the patient’s BSA, and the choice of the Smartcannula was based on their height. *Results*: There were no significant differences between the two groups in terms of negative pressure applied, target flow achieved, hemolysis, the need for blood transfusion, and the post-operative increases in liver and renal enzymes. However, in all the patients, the estimated target flow was achieved, thereby showing the better hemodynamic performance of the virtually wall-less cannula, since, in this group, the patients’ BSA was significantly greater compared to the thin-wall group. Ultimately, the mean cross-clamp time, as an indirect index of the effectiveness of the venous drainage, is shorter in the virtually wall-less group compared with the thin-wall group. *Conclusions*: The virtually wall-less cannula should be preferred in minimally invasive mitral valve surgery due to its superior performance in terms of venous drainage compared with the standard thin-wall cannula.

## 1. Introduction

MICS has been developing since 1996, when Alain Carpentier performed the first right mini-thoracotomy for a mitral valve replacement [1].

In general, inadequate venous drainage during open heart surgery can disturb the physiological flow of the blood, caused by intermittent atrial and/or caval collapse, leading to complete shut-off of the venous drainage, and the blood trapped in the pulmonary circulation floods into the surgical field.

Several factors can influence the quality of venous drainage during cardiopulmonary bypass (CPB), including pre-load, after-load, pump, and venous cannula designs [2]. Indeed, the design, positioning, and choice of venous cannula are probably the most important factors, all conditioning the quality of the venous drainage.

Good venous drainage and a bloodless field during MICS are crucial for the success of an operation, and this is especially true in MICS, where peripheral cannulation is required but more difficult to achieve [3].

The aim of our study was to compare venous drainage using two different types of cannulas in minimally invasive mitral valve surgery, using the virtually wall-less cannula vs. standard thin-wall venous cannula, especially when the size of the venous tube of the CPB circuit is smaller than standard.

## 2. Materials and Methods

This study was retrospective and observational. We evaluated 65 consecutive elective patients who were undergoing MICS for isolated mitral valve disease from January 2021 to December 2022.

Exclusion criteria were age (<18 years), urgency/emergency, moderate aortic valve regurgitation, ascending aorta diameter (>40 mm), and prior right lung pleuritis and/or surgery. Informed consent was obtained from all subjects involved in the study, and our research was performed in accordance with the Declaration of Helsinki.

In our Cardiac Division, there is a dedicated minimally invasive team, and the first surgeon was the same for all of the patients.

We used either a virtually wall-less cannula or a thin-wall cannula inserted into the common femoral vein for venous drainage.

The choice of the type of cannula used was based on both the height and body surface area of the patient, thus allowing us to always maintain our surgical setting of double venous cannulation.

A centrifugal pump (HL20 Heart-lung Machine, Maquet Getinge Group) with a hollow-fiber membrane oxygenator (QUADROX i Adult, Maquet Getinge Group) and a conventional air-oxygen blender were used in all the procedures.

We always performed a CPB with a custom-made circuit (Maquet Getinge Group) with both an arterial and a venous tube of 3/8, thereby reducing the priming volume and the surface of contact with the blood.

The venous reservoir was placed at a level of about 15 cm below the right atrium of the patient. Vacuum pressure was applied to the venous reservoir in order to achieve satisfactory venous drainage.

The internal pressure of the venous reservoir was measured by directly applying a pressure line at the transducer, with each pressure displayed on a central monitor on the console. Inlet and outlet oxygenator pressure were monitored routinely.

After inducing anesthesia, the patient was intubated with a double-lumen endotracheal tube, and was thereby able to exclude to the ventilation of the right lung.

The patient was placed in the usual position, with the right side of the chest elevated at an angle of around 45°.

In all cases, transesophageal echocardiography was used and external defibrillation patches were placed.

The femoral vessels were exposed for cannulation at the level of the right groin.

After administering the heparin (300 UI/Kg), the common femoral artery and the vein were cannulated using the Seldinger technique.

The virtually wall-less cannula (Smartcannula LLC, Lausanne, Switzerland) features a long tubular grid placed inside the inferior vena cava, the right atrium, and the superior vena cava. There is a covered terminal part that is usually out of the femoral vein and connected to the cardiopulmonary circuit.

The introduction of the cannula, both percutaneous and surgical cut-down, requires a soft guidewire introduced in the vein. After enlarging the vein with some progressive dilators, the collapsed cannula is inserted over the wire and positioned at the tip at the origin of the inferior vena cava.

After removing the mandrel, the cannula expands itself up to its standard 24 Fr diameter.

**Figure 1:**
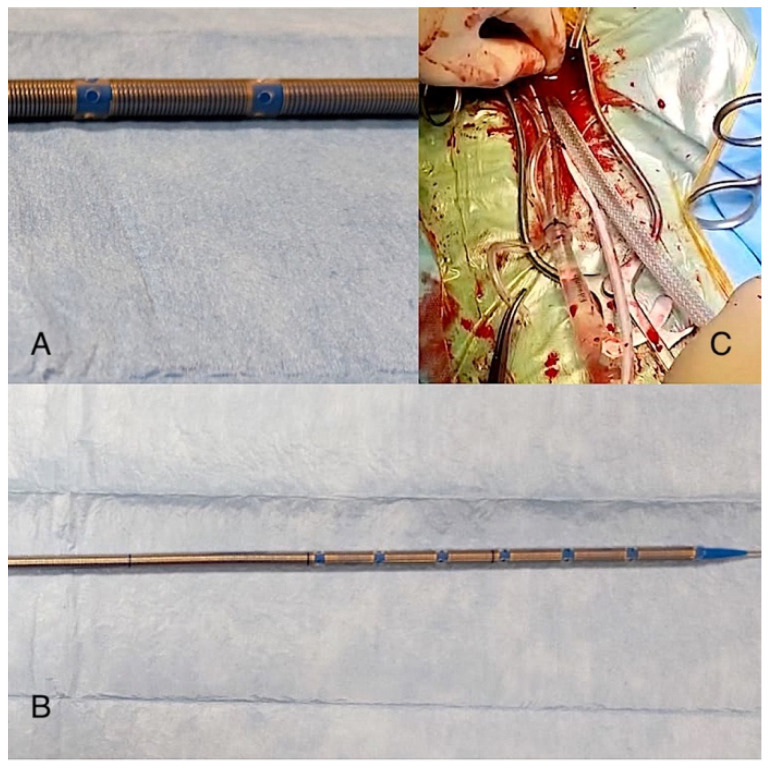
The image shows the Freelife GmbH cannula (**B**) and the particular position and shape of its holes (**A**) compared to the Smartcannula (**C**).

In the standard group, we usually used a thin-wall venous cannula (Freelife Medical GmbH) that has discrete holes in the plastic tubing, which allow for drainage at specific spots (Figure 1).

In the standard group, the size of the cannula was chosen depending on the patient’s body surface area (BSA), while the choice of the Smartcannula was based on their height.

In all cases, we preferred to use a virtually wall-less cannula that was percutaneously inserted into the right jugular vein positioned at the level of the junction between the superior cava vein and the right atrium.

Jugular and femoral venous cannulas are joined to each other with a “Y” connector, which makes up the venous inlet to the cardiopulmonary bypass circuit.

Thus, mildly hypothermic CPB (32 °C) was started.

The gravitational drainage was always combined with vacuum-assisted venous drainage (VAVD), which was progressively increased in order to achieve the maximum active venous return. Afterward, VAVD was slowly decreased to reach a negative pressure always below −40 mmHg.

At this level of negative pressure, a venous collapse was not usually produced, and it allowed an acceptable level of venous drainage to be obtained and achieved the estimated target flow.

After aortic cross-clamping, intermittent antegrade cold blood cardioplegia solution was administered through an aortic root needle.

We performed a mitral valve repair or replacement through a left paraseptal atriotomy.

After sewing the atrium and performing a careful de-airing of the left cardiac chambers, the aortic cross-clamp was removed and cardiopulmonary-weaned.

The removal of the virtually wall-less cannula requires the pinching of the cannula itself at its exit from the femoral vein when pulling out the device. This maneuver allows blood loss to be minimized.

The aim of our study was to compare the performance of the two types of cannula in terms of achieving target flow, venous drainage, and the need to apply negative pressure. Mean cross-clamp time was considered an index of the quality of the bloodless surgical field and the efficiency of venous drainage, which was also evaluated as a reduced increase in liver and renal enzymes as well as the parameters of hemolysis in the first post-operative period.

Continuous variables are expressed as mean and standard deviation, whereas categorical variables are reported as counts and percentages.

Data were analyzed with SPSS 24 (SPSS Inc., Chicago, IL, USA).

The unpaired *t*-test was used to compare all continuous variables between the groups. The changes in AST, ALT, creatinine, and hemoglobin values between the two groups before and after the surgery were assessed with ANOVA for repeated measures. The statistical significance was set with a *p*-value < 0.05.

## 3. Results

The patients’ baseline characteristics are summarized in Table 1.

All patients were successfully discharged from the hospital. The patients were divided into two groups, Group 1 and Group 2, which were the virtually wall-less cannula group and the thin-wall venous cannula group, respectively.

Wall-less venous cannulas measuring either 630 mm (n = 31) or 680 mm (n = 11) in length were successfully introduced in the patients who belonged to Group 1. In only one case did the insertion of the Smartcannula through the common femoral vein cause the perforation of the right appendage, and this was repaired with a little purse string, thereby putting the cannula in the correct position in the atrium.

In the standard group, we used the 24 Fr thin-wall cannula (n = 18) in most of the cases, whereas in very small patients, we preferred the 22 Fr thin-wall cannula (n = 5).

In the virtually wall-less cannula group, the BSAs of the patients were significantly greater compared to the other group.

There were no statistical differences between the groups in terms of hemolysis and the post-operative increases in liver and renal enzymes (Table 2).

Overall, we did not find either intraoperative hemolysis or post-operative signs of liver and renal damage in the two groups.

Pre-operative hemoglobin levels were slightly lower in Group 2 compared to Group 1, although we did not find any statistical differences between them in terms of post-CBP hemoglobin value (*p* = 0.39).

However, during CPB, the mean number of transfused red blood cell units was significantly higher in Group 2 than in Group 1, and this may be a result of the major hemodilution being required to maintain the estimated target flow when we used the thin-wall cannula.

**Figure 2:**
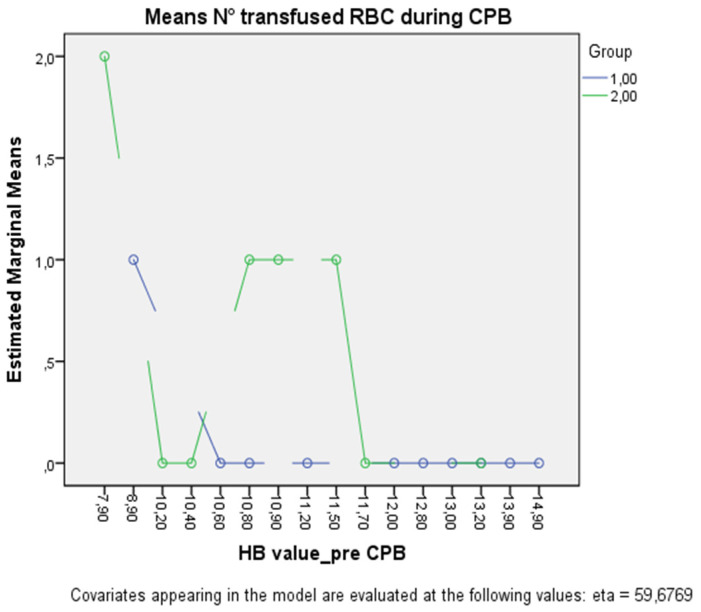
The figure shows the plot separated by group (Group 1, green line; Group 2, blue line) in which each point indicates the estimated marginal mean number of transfused RBCs adjusted for age at each pre-CPB hemoglobin value.

The multivariate ANOVA adjusted for age revealed a significant effect of interaction between the type of cannula and the pre-CPB hemoglobin value, both on the mean number of transfused RBC (*p* < 0.005) and the post-CPB hemoglobin level (*p* < 0.005) (Figure 2).

Additionally, binary logistic regression revealed that older age (OR 1.20 [95% CI, 1.045–1.384] *p* = 0.01) and the use of a standard thin-wall cannula (OR 68.4 [95% CI, 1.43–3289.97] *p* = 0.032) were independent risk factors for the transfusion of one or more units of RBC, whereas the pre-CPB hemoglobin value (*p* = 0.07) and the duration of CPB (*p* = 0.4) were not statistically significant

This consideration would also confirm the better hemodynamic performance of the virtually wall-less cannulas.

There were no differences between the rate of mitral valve repair or replacement in both groups.

Indeed, although only the post-operative peak of aspartate transaminase (AST) was significantly higher compared to the baseline value (*p* = 0.007), these changes were not different in the two groups (*p* = 0.825).

The negative pressure values of VAVD were −31.66 ± 7.52 mmHg and −34.23 ± 5.34 mmHg in the wall-less group and the thin-wall group, respectively (*p* = 0.402).

The estimated target flow was achieved in all patients despite the BSA in Group 1 being significantly greater than in Group 2 (1.84 m^2^ ± 0.14 vs. 1.65 m^2^ ± 0.1; *p* = 0.005)

The mean aortic cross-clamp was shorter in Group 1 than in Group 2 (67.85 ± 9.96 vs. 79.6 ± 13.99 min), likely as a consequence of better exposure due to a more bloodless intracardiac surgical field.

We did not record any neurological complications, conversion to sternotomy, or a need for re-exploration of bleeding.

## 4. Discussion

Traditionally, cardiac surgery was performed through a median sternotomy, which provides generous access to the heart and the great vessels.

In this setting, venous drainage is usually achieved with gravity, and it depends on the difference in height between the right atrium and the venous reservoir.

Since the procedure of minimally invasive cardiac surgery has been developing, venous drainage has become very important.

Indeed, good venous drainage is essential in order to obtain a bloodless surgical field and perform minimally invasive mitral valve surgery.

The performances of venous cannulas keep improving, and this is a result of (and, indeed, reliant on) technological progress. The thin-wall cannulas with multiple side holes are designed to optimize the flow and drainage.

Despite this, during MICS, gravity-assisted venous drainage is often inadequate because of the length and relatively small size of the cannulas.

The VAVD combined with the standard cannula has been used to optimize venous drainage, even if the need to apply excessive negative pressure can often lead to it worsening [2].

Indeed, excessive negative pressure can cause hemolysis and/or the chattering phenomenon due to right atrial collapse around the venous cannula.

VAVD utilizes a standard vacuum suction source connected to a hard-shell venous reservoir, which creates suction in the entire drainage system and increases venous return. This requires close regulation of the vacuum source in order to avoid negative pressure variations.

The excessive VAVD causes trauma to the blood components in addition to the crack or implosion of the venous reservoir.

The applied negative pressure is usually about −40 mmHg. The chattering phenomenon with incomplete venous drainage appears when the pressure is higher than −70 mmHg.

Inadequate venous drainage during MICS not only causes discomfort for the surgeon but also does not allow the estimated target flow to be reached, which is defined as 2.4 lt/min/m^2^.

For these reasons, the virtually wall-less cannula was created as a temporary caval stent to maximize venous drainage, preventing the caval axis from collapsing despite the negative pressure applied by the augmentation through the venous line.

This design, in the same manner as a grid, allows direct venous drainage at all levels of caval affluent vessels, particularly the renal and hepatic veins.

Moreover, the self-expanding characteristic of the wall avoids the caval injury caused by the oversizing of the cannula.

On the contrary, to obtain the necessary seal for drainage with negative pressure, it is necessary to place more than 2 cm of covered wall into the common femoral vein [3].

The original self-expanding cannulas have a nominal diameter of 36 Fr [4], pass through a 24 Fr orifice, fit easily with all venous access diameters from 24 Fr to 36 Fr, and are efficient enough to provide full-flow gravity drainage alone [5].

Despite this concern, it became evident that a smaller-diameter design might eventually work in combination with augmented venous drainage [6].

After various studies both in silico [7] and in vitro [8,9], it has been demonstrated that thinner 24 Fr virtually wall-less cannulas designed for augmented venous drainage seem to function well in combination with additional negative pressure.

Following these positive results, von Segesser et al. found some reasons to extend the use of this cannula in clinical practice, which could be explained by their increased performance designs compared to the traditional thin-wall cannulas [5].

First, the larger mean cross-sectional diameter of the self-expanding design, which is due to the collapsed insertion and expanding in situ approach, achieved an intravascular diameter above the access channel diameter, a feature which, by definition, cannot be achieved with traditional rectilinear cannulas.

Second, the reduced wall thickness of virtually wall-less design typically measures less than 0.5 mm wherever there is a cannula wall akin to the one in the access channel, and this compares to more than 0.5 mm for the standard thin-wall cannula.

Third, the total area of cannula orifices is at least one order of magnitude higher than the one of a typical thin-wall cannula.

Fourth, the virtually wall-less design allows for the direct drainage of blood at all levels of affluent veins compared to the traditional thin-wall cannula (which has only a few holes, located especially in the distal part).

Fifth, the temporary caval stenting function of the virtually wall-less design keeps the drained vessel open over the entire cannula length, and this leads to better drainage in both the experimental [10] and the clinical setting.

As a result of the improved performance, a single virtually wall-less cannula is sufficient for full flow and dual cannulation, and this could be limited in cases in which the right atrium has to be opened, as pointed out by Colangelo et al. [2].

In other experiences, including that of our medical center, surgeons often preferred to perform a dual cannulation using a virtually wall-less cannula, both in the internal jugular and in the common femoral veins.

Another important aspect is the significantly lower negative pressure required for a full flow in combination with virtually wall-less cannula designs per se compared to the standard thin-wall percutaneous cannulas.

Some in vitro studies have demonstrated that the wall-less cannula design performed better than the traditional thin-wall one in a negative pressure situation [11], and that it also showed significantly higher flow rates in water and blood tests [12].

Indeed, Li et al. created a realistic caval tree model and demonstrated that the virtually wall-less one had a better flow rate for each RPM of the pump, with a mean flow increase of about 37% compared with the thin-wall cannulas. In this study, the authors used a wall-less cannula with active drainage by a centrifugal pump. They were able to show in vitro the superiority of the virtually wall-less cannula compared to a control thin-wall cannula.

Berdajs et al. confirmed this suggestion in vivo by demonstrating that with a negative pressure of −43 mmHg, the theoretical blood flow could be reached with 18 Fr access and a wall-less cannula [13]. Using 25 Fr access and a wall-less cannula, Mueller et al. were able to achieve an increase in blood flow of 43% compared to a thin-wall cannula of the same size [14].

The difference in the results between Li and Mueller can be explained by the different sizes of the wall-less cannulas. However, all wall-less cannulas allow a major blood flow to be achieved compared to thin-wall cannulas of any size.

Li et al. observed the different behaviors between the wall-less cannula and the thin-wall cannula in case of aspiration with progressively increased negative pressure. For the 25 Fr thin-wall cannula, the aspiration of the caval wall substitute into the orifices at the cannula tip occurs at a negative pressure >−120 mmHg, while it is a little lower for the 23 Fr thin-wall cannula.

Usually, a complete pump-stop is required to release the grip of the venous wall before the flow can progressively start again, and a balance is achieved between venous drainage and negative pressure below the collapse level.

For the 24 Fr virtually wall-less cannula, the flow of 10 lt/min can be achieved without exceeding −70 mmHg of negative pressure and avoiding caval collapse.

However, other studies with the 24 Fr 530 mm wall-less cannula revealed that, at excessive negative pressure, even if below −120 mmHg, the wall of the cava can progressively collapse over the stent of the self-expandable cannula, leading to an increasing reduction in venous drainage as a consequence of its compression by the same caval wall. The reduction in the level of negative pressure applied allows the re-expansion of the virtually wall-less cannula, thereby recovering the venous drainage [11].

Although some in vitro studies found a correlation between the augmented venous drainage with either a vacuum or a centrifugal pump as well as the incidence of micro-embolism in the arterial line [15,16] at a negative pressure up to −40 mmHg, there were no differences compared to gravity venous drainage, especially in the clinical setting [17,18].

Another important consequence of excessive negative pressure is hemolysis due to mechanical damage to red blood cells.

This concern has proven to be unfounded for the stent-type design of the virtually wall-less cannulas studied here, as demonstrated previously, used in combination with gravity drainage [4,19] and augmented venous drainage [20].

In our experience, the VAVD has been used in both groups without a significant difference in the mean negative pressure required, even if it was slightly lower in the virtually wall-less cannula one, and, still, it was always less than −40 mmHg in all cases.

Moreover, we did not find in either group the onset of either intraoperative hemolysis or post-operative renal, hepatic, or brain damage.

However, changing the point of view, we have been able to demonstrate that the performance of the virtually wall-less cannula was superior compared to the standard thin-wall cannula for two reasons.

First, in the virtually wall-less cannula group, the BSAs of the patients were significantly higher than in the other group. So, achieving the estimated target full flow in this group with a slightly lower additional negative pressure can be only explained as the consequence of better venous drainage due to the design of the cannula. However, in both groups, the mean negative pressure is lower than −40 mmHg, thereby avoiding its negative effects in terms of hemolysis and worsening venous drainage due to the chattering phenomenon.

Second, even in larger patients, we found that the virtually wall-less cannula allows a more bloodless surgical field to be obtained compared with the standard cannula, leading to a significant reduction in aortic cross-clamp time and major satisfaction on the part of the cardiac surgeon.

The lesser reduction in the mean additional negative pressure in comparison with the data published in the literature probably depends on the small size of the venous line (3/8″), which we preferred to use, thereby reducing hemodilution due to the priming.

## 5. Limitations

This study has some limitations. Our results could be underestimated by the small size of the sample, the prevalent use of virtually wall-less cannulas, and the size of the venous line of our cardiopulmonary bypass circuit.

The choice between the virtually wall-less cannula and the thin-wall cannula was often based on the BSA and height of the patients, and thus, the thin-wall cannula was mainly used in smaller patients. This choice could have minimized the difference in the required negative pressure between the groups.

## 6. Conclusions

We strongly believe in MICS for its cosmetic advantages and the faster recovery of patients.

Good venous drainage and a bloodless field during MICS are both crucial for the success of the operation.

In order to achieve these results, the virtually wall-less cannula should be preferred in minimally invasive mitral valve surgery, especially in overweight patients, due to its superior performance in terms of venous drainage compared with the standard thin-wall cannula.

## Figures and Tables

**Table 1:** The patients’ baseline characteristics.

	Virtually Wall-Less Cannula (Group 1—N = 42)	Standard Thin-Wall Cannula (Group 2—N = 23)	*p* Value
Age (y)	59.16 ± 10.23	60.5 ± 17.77	0.91
Female [N (%)]	24 (57%)	10 (43.4%)	0.19
B.S.A. (m^2^)	1.84 ± 0.14	1.65 ± 0.1	0.01
E.F (%)	60.5 ± 2.84	60.7 ± 11.01	0.96
Hb value (g/dL)	12.15 ± 1.96	10.52 ± 1.38	0.06
AST (UI/L)	22.92 ± 4.63	32.4 ± 17.42	0.08
ALT (UI/L)	54.77 ± 23.54	61.6 ± 18.2	0.47
Creatinine value (mg/dL)	0.82 ± 0.14	0.82 ± 0.6	0.99
Mitral valve disease [N (%)]			
Rheumatic Barlow/FED	19 (45.3) 23 (54.7)	13 (56.6) 10 (43.4)	0.11

B.S.A.: body surface area; ALT: alanine transaminase; AST: aspartate transaminase; Hb: hemoglobin value; FED: fibroelastic disease.

**Table 2:** Patients’ intra-operative and post-operative parameters.

	Virtually Wall-Less Cannula (Group 1—N = 42)	Standard Thin-Wall Cannula (Group 2—N = 23)	*p* Value
Hb pre-CPB (g/dL)	12.15 ± 1.96	10.52 ± 1.38	0.06
Hb post-CPB (g/dL)	10.847 ± 1.58	10.18 ± 0.7	0.39
Transfusion * (U)	0.08 ± 0.29	0.67 ± 0.82	0.04
AST (UI/L—peak)	22.92 ± 4.63	32.4 ± 17.42	0.08
ALT (UI/L—peak)	54.77 ± 23.54	61.6 ± 18.2	0.47
Creatinine value (mg/dL—peak)	0.82 ± 0.14	0.82 ± 0.6	0.99
Aortic cross-clamp time (min)	67.85 ± 9.96	79.6 ± 13.99	0.04
Target flow (L/min)	4.04 ± 0.31	3.64 ± 0.22	0.01
LDH (UI/L—peak)	288.46 ± 66.6	335.25 ± 81.98	0.35
MV rep [N (%)] Ring & res P2 Ring & e-to-e	23 (54.7) 13 10	10 (43.4) 6 4	0.11
MVR [N (%)]	19 (45.3)	13 (56.6)	

LDH: lactate dehydrogenase; ALT: alanine transaminase; AST: aspartate transaminase; Hb: hemoglobin value; (*): mean number of transfused red blood cell units during CPB; MV rep: mitral valve repair; MVR: mitral valve replacement; Ring & res P2: annuloplasty ring and quadrangular resection P2; Ring & e-to-e: annuloplasty ring and edge-to-edge.

## Data Availability

The data that support the findings of this study are not openly available because they are human data and are available from the corresponding author upon reasonable request.
